# Preparation of sea buckthorn (*Hippophae rhamnoides* L.) seed meal peptide by mixed fermentation and its effect on volatile compounds and hypoglycemia

**DOI:** 10.3389/fnut.2024.1355116

**Published:** 2024-02-13

**Authors:** Jiangyong Yang, Jingyang Hong, Aihemaitijiang Aihaiti, Ying Mu, Xuefeng Yin, Minwei Zhang, Xiaolu Liu, Liang Wang

**Affiliations:** College of Life Science and Technology, Xinjiang University, Urumqi, China

**Keywords:** sea buckthorn seed meal, fermentation, volatile compounds, peptides, hypoglycemic

## Abstract

This study employed mixed bacterial strains to ferment seabuckthorn seed meal into peptides, and conducted a comprehensive evaluation of the growth adaptive conditions, molecular weight distribution, volatile compounds, and *in vitro* hypoglycemic activity required for fermentation. Results showed that when the amount of maltose was 1.1% and MgSO_4_·7H_2_O was added at 0.15 g/L, the peptide yield reached 43.85% with a mixed fermentation of *Lactobacillus fermentum, Bacillus subtilis, Lactobacillus casei, Lactobacillus rhamnosus*, and *Lactobacillus acidophilus*. Components with a molecular weight below 1 kDa were found to be more effective in inhibiting the activity of α-amylase and α-glucosidase, with the identified sequence being FYLPKM. Finally, SPME/GC–MS results showed that 86 volatile components were detected during the fermentation of seabuckthorn seed meal, including 22 alcohols, 9 acids, 7 ketones, 14 alkanes, 20 esters, and 14 other compounds. With prolonged fermentation time, the content of acids and esters increased significantly.

## 1 Introduction

Sea buckthorn (*Hippophae rhamnoides* L.) is a small deciduous tree or shrub in the family Elaeagnaceae, also known as the vinegar tree fruit. It is rich in nutritional value and is used as a medicinal and edible plant (1). The stem, leaves, berries, and seeds of sea buckthorn all contain abundant vitamins, fatty acids, amino acids, flavonoids, superoxide, and other bioactive substances. These bioactive substances have significant effects such as lowering blood pressure, anti-inflammatory, anti-diabetic, and preventing cardiovascular diseases (2). Sea buckthorn seed meal is a by-product of extracting oil from sea buckthorn seeds. It has a protein content of over 35% and a complete range of amino acids, making it an important source of complete protein (3). However, sea buckthorn seed meal is usually used as animal feed or directly discarded, leading to serious waste of protein resources.

Bioactive peptides are generally released from proteins through proteolysis or fermentation, and they have broad biological activities and pharmacological properties, including anti-tumor activity, improvement of memory, and regulation of metabolic disorders (4). Therefore, they have broad application prospects in health products, food preservation, and pharmaceutical engineering (5). From the perspective of sustainable development and utilization of biological resources, the protein resources in sea buckthorn seed meal have enormous potential in the production of bioactive peptides.

Currently, the main methods for preparing bioactive peptides include enzymatic hydrolysis, microbial fermentation, chemical hydrolysis, and genetic engineering (6). Most peptides are prepared by enzymatic hydrolysis, and there is limited research on the direct fermentation for peptide production. Enzymatic hydrolysis has strong specificity and high extraction rate, but it is costly and not suitable for large-scale production. However, microbial fermentation can effectively address this issue. Fermentation has low costs, can convert substrates into beneficial bioactive substances, and allows for the production of unique products (7). Research have shown that *Lactobacillus thermophilus* can be used for fermentative production of whey protein peptides, which exhibit high ACE inhibition and antioxidant activity (8). *Bacillus* fermentation of coffee grounds can increase the content of protein hydrolysates, leading to a significant increase in soluble protein and small peptide content (9). *Lactobacillus plantarum* fermentation of almonds can degrade the molecular weight of almond proteins, altering the external morphology of the proteins and impacting their immunoreactivity (10). Additionally, microbial fermentation not only preserves nutritional components but also generates new volatile compounds through microbial metabolism, enhancing the aroma of the product (11). Different strains have different effects on the bioactivity and flavor of the fermentation substrate. For example, *Lactococcus lactis* can better utilize carbon sources for proliferation, increase the production of organic acids, and effectively improve the bitterness and astringency of sea buckthorn juice (12). *Lactobacillus plantarum* can synthesize extracellular polysaccharides with blood glucose-lowering activity through its glycoside hydrolase activity (13). Furthermore, compared to single-strain fermentation, mixed-strain fermentation can not only increase the variety and content of volatile flavor compounds but also enhance the antioxidant activity of the fermentation substrate (14). However, bioactive peptides as a functional food have not been well commercialized yet. Therefore, the fermentation-based preparation of sea buckthorn seed meal peptides and exploration of changes in volatile compounds and *in vitro* hypoglycemic activity in fermented peptide solutions are of significant importance for the reutilization of sea buckthorn seed meal and its future food applications. This study employed a mixed-strain fermentation method and screened out 5 strains with the best fermentation effect using peptide yield to degrade protein from sea buckthorn seed meal. The peptide molecular weight and amino acid content of samples at different fermentation times were detected and analyzed to determine the optimal conditions for fermentation peptide preparation. Then, we investigated the inhibitory activity of components with different molecular weights on α-amylase and α-glucosidase, and sequenced the best components. Finally, HS-SPME-GC–MS was used to detect the dynamic changes in volatile compounds during the fermentation process of sea buckthorn seed meal, providing a standard for its edibility, contributing to reducing waste of sea buckthorn protein resources and increasing the added value of the products.

## 2 Materials and methods

### 2.1 Materials and instruments

The sea buckthorn seed meal (protein: 45%) was obtained from Xinjiang, China, and all chemicals had analytical purity or better. The water bath and incubator were purchased from Beijing Yong Guangming Medical Instrument Co. (Beijing, China) and Shanghai Qixin Scientific Instrument Co. (Shanghai, China), respectively. The UV–Vis spectrophotometer and electrophoresis instrument were obtained from Mpoda Instruments Ltd. (Shanghai, China) and Beijing Liuyi Instrument Factory (Beijing, China), respectively. The enzyme labeler and centrifuge were purchased from Molecular Devices (California, United States) and Jiangsu Jinyi Instrument Technology Co. (Jiangsu, China), respectively. The microscope was from Motic (Fujian, China). Amylase (3,800 U/g) and lipase (100,000 U/g) were obtained from Beijing Aobo Star Biotechnology Co. (Beijing, China) and Shandong Longke Keto Enzyme Preparation Co. (Shandong, China), respectively. Ten species of commercial strains *Lactobacillus delbrueckii subsp. Bulgaricus* CICC6098 (*L. bulgaricus*), *Bifidobacterium animalis subsp. lactis* CICC21712 (*B. lactis*), *Lacticaseibacillus paracasei* CICC6237 (*L. paracasei*), *Lactococcus lactis subsp. Lactis* CICC23196 (*L. lactis*), *Lactobacillus acidophilus* CICC6086 (*L. acidophilus*), *Limosilactobacillus fermentum* CICC25124 (*L. fermentum*), *Limosilactobacillus reuteri* CICC6123 (*L. reuteri*), *Lacticaseibacillus case* CICC6114 (*L. case*), *Lactipalntibacillus plantarum* CICC25125 (*L. plantarum*), *Bacillus subtilis* CICC24713 (*B. subtilis*) were provided by the China Industrial Microbial Strain Preservation and Management Centre (Beijing, China).

### 2.2 Selection of culture media

The sea buckthorn seed meal had a material-to-water ratio of 1:34 (W/V). The bacterial density was used as an indicator. The activated 1 × 10^7^ CFU/mL *L. casei* was inoculated in the aqueous solution of the sea buckthorn seed meal, followed by the addition of 2% glucose, maltose, sucrose, lactose, and starch as carbon sources to the medium for the one-factor experiments on the basis of MRS to determine the carbon source, and 0.5, 0.8, 1.1, 1.4, 1.7, and 2% of the carbon sources for the one-factor experiments. Factorial experiment. Based on the previous step, different concentrations of FeSO_4_·7H_2_O, ZnSO_4_·7H_2_O, CuSO_4_·5H_2_O, MgSO_4_·H_2_O and NaCl trace elements were added to determine the fermentation conditions of the sea buckthorn seed meal in one-way experiments.

### 2.3 Preparation of the fermented peptide

The aqueous solution of the sea buckthorn seed meal was pre-cooked with water for 30 min at 85°C and cooled. Based on the weight of the sea buckthorn seed meal powder, the reaction was terminated by adding 0.2% α-amylase and 0.4% lipase for 5 h in a water bath at 50°C. The reaction was then inactivated at 85°C for 30 min. After inactivation of the reaction, the sea buckthorn seed meal solution was cooled, and based on an initial bacterial density of 1 × 10^7^ CFU/mL, 10 fermentation strains were separately added to the seed meal solution. Following inoculation, the aqueous solution of the sea buckthorn seed meal was fermented statically at 37°C. The peptide content was detected every 4 h, and the fermentation was terminated when the peptide content was stable.

### 2.4 Measurement of the peptide content

According to the method of Yan et al. (15), 1 mL sample solution was collected. Then, 1 mL of 15% (W/W) trichloroacetic acid (TCA) aqueous solution was added to the solution, mixed, left to stand for 8 min, and centrifuged at 4,000 r/min for 10 min. Subsequently, 1 mL of the supernatant was collected and 4 mL of bis(ureido)urea reagent was added to the supernatant. Similarly, the solution was mixed and left to stand for 25 min. At the same time, l mL of water and 4 mL of bis(ureido)urea reagent were mixed as a blank. Absorbance values were determined at 540 nm. The peptide concentration C (mg/mL) in the hydrolysate was calculated using a standard regression equation, and the peptide yield was the percentage of soluble peptides obtained through fermentation in the total protein.

### 2.5 Determination of protein hydrolysis

Following the determination of the fermentation time for each strain, the time point with the highest peptide yield was selected, and the fermentation residue was dried. And the nitrogen content was detected using the Kjeldahl method GB5009.5-2016. The protein conversion coefficient was 6.25, and the hydrolysis degree of the sea buckthorn seed meal fermented by different strains was calculated as follows:


$$D\left(H\right)=\frac{N-N1}{N}\times 100\%$$

Note: *N*1 is nitrogen in the fermentation residue, *g*; *N* is total nitrogen in the feedstock, *g*.

### 2.6 Unified design to determine the proportion of mixed bacteria

Based on the experimental results of the peptide content and hydrolysis degree, 5 dominant strains were selected from among the 10 fermentation strains for mixed fermentation. The optimization experiments were designed using a uniform design table with 5 dominant strains, 10 factors as levels, 19 h as fermentation time and peptide yield as the response value. Quadratic polynomial stepwise regression analyses were conducted using SPSS 20.0 software to obtain quadratic polynomial regression equations. The programmed solver function in Excel was then used to solve the problem and determine the optimal fermentation inoculation ratio.

### 2.7 Sodium dodecyl sulfate-polyacrylamide gel electrophoresis

We then conducted SDS-PAGE analyses of sea buckthorn seed meal solutions fermented at different times by mixed bacteria and the optimal fermentation time of the samples was preliminarily determined using the electrophoretic conditions of Fan et al. (16).

### 2.8 Molecular weight distribution

The main operating parameters were as follows:

Column: TSKgel 2000 SWXL 300 mm × 7.8 mm; mobile phase: acetonitrile/water/trifluoroacetic acid, 45/55/0.1 (V/V); detection: UV 220 nm; flow rate: 0.5 mL/min; column temperature: 30°C.

Sample preparation: 100 mg of the sample was placed in a 10-mL volumetric flask, diluted proportionally with the mobile phase, and filtered using a 0.45-μm microfilm.

Sample solutions with different fermentation times were analyzed under the aforementioned chromatographic conditions, and the data were processed using GPC software to obtain the peptide phases of the samples, and paired molecular mass distributions and their distribution ranges.

### 2.9 Detection of amino acid content

First, 2 mL of each sample was added to a 10-mL glass tube. Then, 2 mL of concentrated hydrochloric acid (containing 1% phenol) was added to the tube, followed by the addition of nitrogen for 1 min. The bottle was sealed, and hydrolysis was allowed to occur at 110°C for 22 h. The solution was removed, cooled, and diluted with water to 50 mL. Then, 1 mL of the solution was blown dry at 95°C under a nitrogen atmosphere, and 1 mL of 0.01 M HCl was accurately added to dissolve the mixture and measured over a membrane.

Mobile phase A: 40 mM sodium dihydrogen phosphate (pH 7.8); mobile phase B: acetonitrile/methanol/water = 45/45/10.

An Agilent transparent ODS column (5 μL, 4.0 mm × 250 mm) was used to detect the amino acid content. The gradient elution was performed using the following elution program: 0 min 0% B; 23 min 57% B; 27 min 100% B; 40 min 0% B. The flow rate of the mobile phase was 1.0 mL/min, and detection was performed using an ultraviolet detector (VWD). The wavelength: 338 nm, proline: 266 nm. The amino acid content was quantified using the external standard method.

### 2.10 Isolation of peptides from the sea buckthorn seed meal

The samples were initially purified using macroporous resin DA201-C, and the fermentation broth was ultrafiltered using ultrafiltration centrifuge tubes with cut-off values of 1 and 3 kDa, respectively, to obtain peptides of 0–1, 1–3, and >3 kDa. These peptides were then lyophilized and stored at −80°C until use.

### 2.11 Detection of α-glucosidase and α-amylase inhibition rates

According to the method of Sadeghi et al. (17), with modification, 30 μL of 5 mg/mL sample solutions of different molecular weights were taken and mixed with 30 μL of α-glucosidase (1 U, dissolved in 0.1 mol/L PBS, pH 6.8). Then, 120 μL of phosphate buffer was added, and the reaction mixture was incubated in a 96-well plate for 25 min at 37°C. Subsequently, 60 μL of pNPG solution was added. The reaction was terminated by adding 70 μL of sodium carbonate solution (0.8 mol/L). The absorbance of the sample was measured at 405 nm. Acarbose was used as a positive control, and an equal volume of phosphate buffer (0.1 mol/L, pH 6.8) was used as a control instead of α-glucosidase solution.


(1) $$\mathrm{Inhibition}\;\left(\%\right)=1-\frac{A1-A2}{A3-A4}\times 100\%$$

Note: A1: absorbance of sample; A2: absorbance of sample control; A3: absorbance of the blank group; A4: absorbance of the blank control.

Referring to and modifying the method of Huang et al. (18). 10 μL of α-amylase solution (10 U/mL) was mixed with 10 μL of sample solution of different molecular sizes. The mixture was incubated at 37°C for 10 min in a water bath, followed by the addition of 500 μL of 0.9% soluble starch solution. The mixture was then incubated at 37°C for 15 min, and 500 μL of DNS reagent was added and placed in a boiling water bath for 8 min to stop the reaction. After cooling, 4 mL of distilled water was added for dilution. Then, 250 μL was transferred to a 96-well plate, and the absorbance was measured at a wavelength of 540 nm. Acarbose was used as a positive control, and the α-amylase enzyme inhibition rate was calculated according to Formula (1).

### 2.12 Sequence identification of peptides

According to the method of Heymich et al. (19), with modifications, the amino acid sequences of the peptides were determined using LC–MS (Thermo Fisher Scientific, United States). A C18 (Acclaim PepMap RSLC, 75 μm × 25 cm, 2 μm, 100 A) reverse-phase chromatography column was used, with the mobile phase B increasing from 5 to 38% within 30 min. Mass spectrometry was performed using the ThermoFisher Q Exactive system (ThermoFisher, United States) combined with a nanospray Nano Flex ion source (ThermoFisher, USA), with a voltage of 1.9 kV, a heating temperature of 275°C, a scan range of 100–1,500 *m/z*, and a collision energy set at 28 eV. Data processing and analysis were conducted using PEAKS Studio 8.5 software (Bioinformatics Solutions Inc., Waterloo, Canada). The biological activity of the peptides was predicted using the online analysis tool.^1^ Additionally, the online analysis tool^2^ was used to analyze the predicted toxicity, hydrophilicity, and hydrophobicity of the peptides.

### 2.13 Analysis of volatile compounds

Referring to and modifying the method of Shi et al. (20). The internal standard was decanol diluted 1,000 times, and samples at different fermentation times were processed. Thermo Fisher Scientific (Waltham, Massachusetts, United States) GC–MS instrument was used to detect volatile compounds in sea buckthorn seed meal. Helium was used as the carrier gas with a flow rate of 1 mL/min, an initial temperature of 40°C, and a hold time of 2 min. The temperature was then increased by 3°C/min to 150°C and held for 4 min, followed by an increase of 10°C/min to 230°C and held for 4 min. The volatile compounds were separated on an HP-5MS chromatographic column (30 m × 250 m × 0.25 um). The ion source temperature was 230°C, and the electron energy was 70 ev. Data were collected at a scan rate of 1/scan within the range of 40–500 *m/z*.

### 2.14 Statistical analysis

All results are presented as mean ± standard deviation (S.D.). Statistical significance (*p* < 0.05; Duncan test) was determined using SPSS 20.0 software (SPSS Science, Chicago, Michigan, United States). Graphs were created using Origin 2021 software (OriginLab, Northampton, MA, United States).

## 3 Results and discussion

### 3.1 Colony density

**Figure 1:**
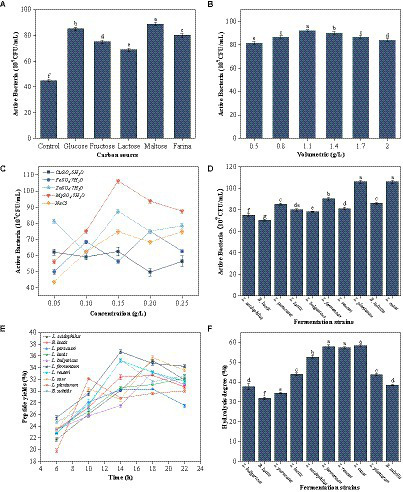
Screening of fermentation strains. **(A)** Effect of different types of carbon sources on viable counts of *L. casei.* **(B)** Effect of different mass concentrations of maltose on viable counts of *L. casei*. **(C)** Effect of different mass concentrations of Cu^2+^, Fe^2+^, Zn^2+^, Mg^2+^ and Nacl on viable counts of *L. casei.* **(D)** Colony densities of different strains of bacteria after media optimisation. **(E)** Changes in peptide yield (%) during fermentation of sea buckthorn seed meal by different strains. **(F)** Results of hydrolysis degree (%) in fermentation residues of different strains (these values are expressed as mean of three replicates ± standard deviation (SD), lower case letters represent significance, *p* < 0.05).

The protein content is relatively high in seabuckthorn seed meal, and microbial growth and metabolism are slow. Therefore, some nutrients need to be supplemented to promote microbial growth. Carbon is a nutritional factor necessary for microbial growth and reproduction. It can regulate the growth and metabolism of microbes and their metabolic properties. It has a direct impact on the fermentation process (21). Among the 5 carbon sources, maltose exerted the most significant effect on the growth and reproduction of *L. case* (Figure 1A). After the addition of different maltose concentrations, the colony density first increased gradually and reached the maximum amount of maltose added was 1.1% (Figure 1B). Then, the colony density exhibited a decreasing trend. Therefore, the optimal amount of the carbon source was 1.1%.

Trace elements promote microbial growth, enhance probiotics activity, change their physicochemical properties, and are a constituent of certain active substances (22). The promoting effect of adding NaCl, Cu^2+^, Zn^2+^, and Fe^2+^ on *L. case* growth and reproduction was not obvious (Figure 1C). By contrast, the promoting effect of MgSO_4_·7H_2_O addition on *L. case* growth was obvious. The colony density reached the highest when the amount of trace elements (e.g., MgSO_4_·7H_2_O) added was 0.15 g/L (Figure 1C) Therefore, the optimal concentration of trace elements to be added was 0.15 g/L.

Additionally, under the same conditions, the colony density of nine other fermentation strains was measured (Figure 1D). It was observed that after the addition of carbon sources and trace elements, the colony density of these 9 strains was all above 6.5 × 10^8^ CFU/mL, indicating that their growth and reproduction were promoted to varying degrees under these conditions.

### 3.2 Determination of peptide content

Different fermentation strains had different fermentation effects (Figure 1E). *L. fermentum* had the best fermentation effect among the 10 strains, with the highest polypeptide production rate being 36.75% ± 0.1%. *L. paracasei* had the worst fermentation effect, with the polypeptide production rate being 30.34% ± 0.03%. Most strains exhibited a trend of increase in the polypeptide production rate and then a decrease in the rate with an increase in fermentation time. The fermentation trend of *L. casei*, *L. plantarum*, and *B. subtilis* fluctuated, exhibiting an increase, then a decrease, followed by a slow increase (Figure 1E). However, the polypeptide production rate did not exceed the peak value in the subsequent fermentation time. The rate of peptide production from *L. lactis* exhibited a trend of slow increase, but the peptide yield was low. The peptide yield of the sea buckthorn seed meal solution fermented by *L. fermentum*, *L. reuteri*, and *B. subtilis* reached a peak at 14 h. A flat or decreasing trend was noted after the peak was attained. By contrast, the polypeptide production rate of the sea buckthorn seed meal solution fermented by *L. acidophilus*, *B. lactis*, *L. paracasei*, *L. bulgaricus*, and *L. casei* increased gradually as the fermentation time was prolonged, and reached the peak value at 18 h. The polypeptide production rate gradually decreased after 18 h, possibly because different strains of microorganisms metabolize and secrete different proteases, which act on the different cleavage sites of the proteins present in the sea buckthorn seed meal, producing peptide fragments with different molecular weights and sizes (23). Thus, the optimal fermentation times are different for different strains and would produce the highest peptide yield. By analyzing the data, we noted that *L. fermentum*, *L. casei*, and *L. acidophilus* produced the best polypeptide yield. The optimal fermentation time was between 14 and 19 h.

### 3.3 Measurement of protein hydrolysis

The fermentation residue at the highest peptide yield during fermentation by the 10 strains was used to determine nitrogen content and thus verify the hydrolysis degree of the sea buckthorn seed meal protein during fermentation. The hydrolysis degree of the sea buckthorn seed meal fermentation broths was >30% in all cases (Figure 1F). Ma et al. (24) performed microbial fermentation of chickpea proteins and examined their nitrogen content after fermentation. The hydrolysis degree was >60% in all cases. This phenomenon may be a result of the high protein hydrolysis capacity of lactic acid bacteria, which have a wide range of adaptability to various environments, inducing secondary protein hydrolysis and enriching them with bioactive peptides. Hou et al., revealed that fermentation can increase the degree of protein hydrolysis, and fermented proteins have better bioactivity (25). Based on the peptide yield and the hydrolysis degree, 5 dominant strains were screened, namely *L. fermentum*, *B. subtilis*, *L. casei*, *L. reuteri*, and *L. acidophilus*.

### 3.4 Homogeneous design determines the proportion of strains

Mixed fermentation has better growth dynamic characteristics, which can improve the quality of the fermented material and reduce the off-flavor (26). According to Peng et al. (27) mixed fermentation can enrich the taste of soymilk and improve the flavor characteristics of soymilk. The proteins in soymilk can be quickly and effectively hydrolyzed into peptides and amino acids to improve their nutritional value. Therefore, the uniform design experiment was used in this study and the results are presented in Table 1. The peptide production rate was used as the response value and the fermentation time was 19 h to determine the ratio between different bacterial strains. A quadratic polynomial stepwise regression equation was established as follows:


$$\begin{array}{l} Y=6.584-30.371X_{1}+36.148{X_{1}}^{2}+0.873X_{3}X_{4}\\ \;+2.278X_{4}X_{5}-0.335X_{2}X_{4}-1.669{X_{5}}^{2}\text{.} \end{array}$$

**Table 1:** Experimental design of strain proportions.

No.	*L. fermentum* X1 (%)	*B. subtilis* X2 (%)	*L. casei* X3 (%)	*L. reuteri* X4 (%)	*L. acidophilus* X5 (%)	Peptide yields (%)
1	36	31	21	16	1	31.52
2	37	32	22	17	2	28.06
3	38	33	23	18	3	28.44
4	39	34	24	19	4	32.01
5	40	35	25	20	5	33.54
6	41	36	26	21	6	33.53
7	42	37	27	22	7	42.68
8	43	38	28	23	8	38.85
9	44	39	29	24	9	30.26
10	45	40	30	25	10	33.10

The regression equation was *R*^2^ = 0.99. The final optimal mix of bacteria was noted to be *L. fermentum* 25.53%, *B. subtilis* 28.36%, *L. casei* 21.27%, *L. reuteri* 17.73%, and *L. acidophilus* 7.11%. The predicted peptide yield at this point was 47.44%. A validation test conducted based on the predicted optimal conditions provided a peptide yield of 43.85% after the fermentation of the sea buckthorn seed meal. This indicates that the equation can predict the optimal ratio well, which confirms the validity of the model.

### 3.5 SDS-PAGE

**Figure 2:**
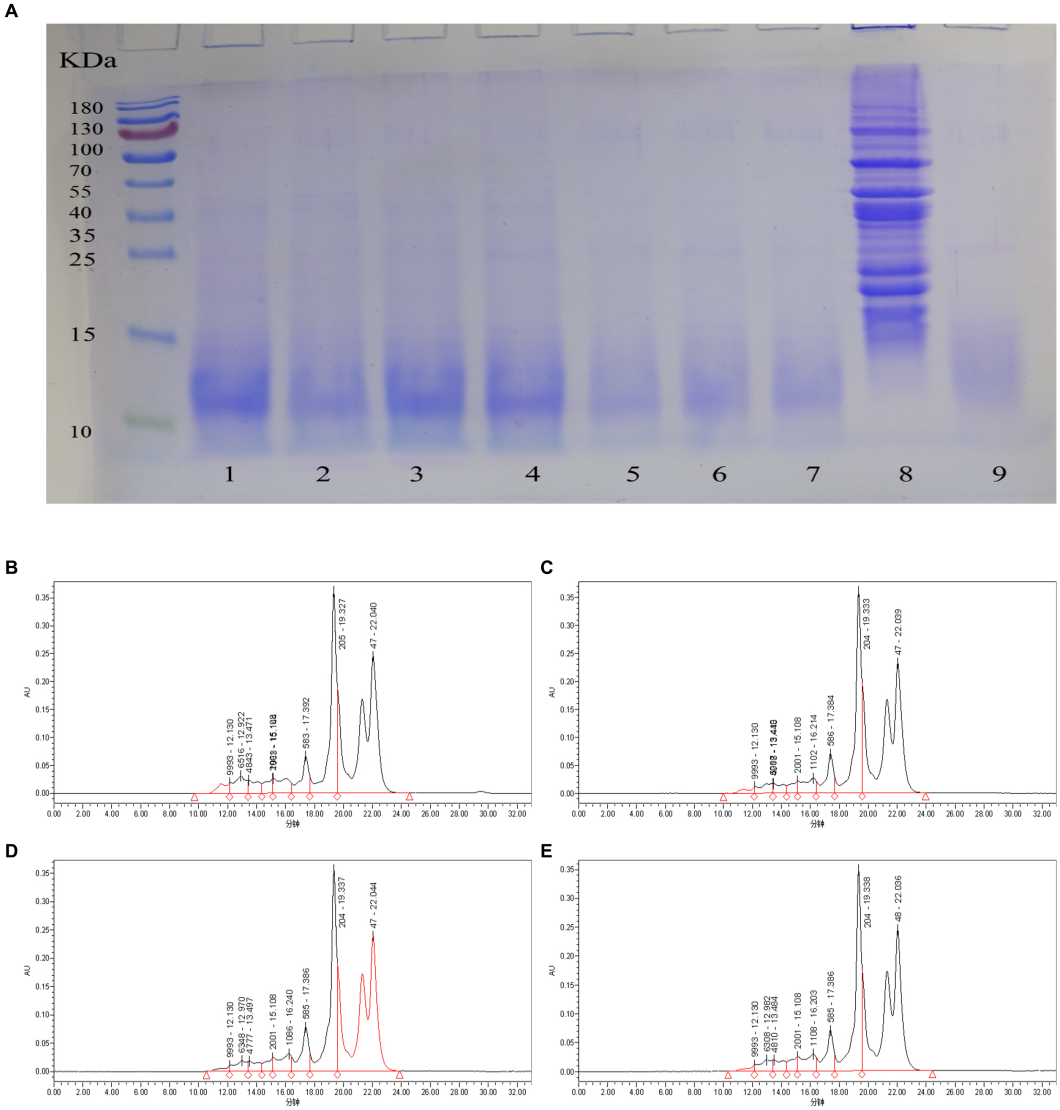
Degree of protein degradation of sea buckthorn seed meal at different times. **(A)** SDS-PAGE of different fermentation times. 1: 10 h, 2: 12 h, 3: 14 h, 4: 16, 5: 18 h, 6: 20 h, 7: 22 h, 8: Raw material, 9: 24 h; **(B)** Molecular weight distribution of peptides at 17 h; **(C)** Molecular weight distribution of peptides at 18 h; **(D)** Molecular weight distribution of peptides at 19 h; **(E)** Molecular weight distribution of peptides at 20 h.

The peptide solution of the sea buckthorn seed meal fermented for different fermentation times was analyzed through SDS-PAGE. The mixed strain fermentation is favorable for the degradation of large-molecular-weight proteins (Figure 2A). The mixed strains can degrade large-molecular-weight proteins into small-molecule peptides and increase the peptide yield. This result is in agreement with Wang et al. (28) who used mixed strains of bacteria to ferment soybean meal to improve its nutritional value. The molecular weight of most proteins in the raw material was distributed in the range of 15 and 130 kDa (Figure 2A). As the fermentation time was prolonged, the large molecules of proteins in the fermentation broth were gradually degraded to <10 kDa. At 10–16 h of fermentation, lactic acid bacteria degraded the macromolecular proteins in the raw material to <15 kDa. However, two shallow bands were still seen above and below 25 and 35 kDa, respectively, indicating that the macromolecular proteins were not completely degraded. After 18–24 h of fermentation, at >10 kDa, the bands were no longer seen on the electrophoresis graph. This indicates that the large molecules of protein were degraded. The bands at approximately 10 kDa were also relatively shallow, which indicated that the mixed strain fermentation had a good decomposition effect on the sea buckthorn seed meal protein. We preliminarily concluded that from 18 h onwards, the macromolecular proteins in the raw material were almost completely degraded to <10 kDa. Thus, the optimal fermentation time of sea buckthorn seed meal peptides was further analyzed.

### 3.6 Molecular weight distribution

The molecular weight distributions of sea buckthorn seed meal peptides at different fermentation times were relatively similar (Table 2). Most of them were distributed in the <1 kDa range. The >10 kDa fraction was degraded to 2.3% at 17 h (Figure 2B), and as the fermentation time was prolonged, the fraction was reduced to 0.91% at 20 h (Figure 2E) This indicates that large-molecule proteins are gradually degraded into small-molecule peptides during fermentation. The 3–10 kDa fraction was decreased to 5.26% at 18 h (Figure 2C), and then, a small recovery to 5.97% was noted at 20 h. The 1–3 kDa fraction decreased to 6.7% at 18 h, and then gradually increased to 7.43% at 20 h, which may be the result of a decrease in the 1–10 kDa fraction to 7.43%. This result was possibly observed because, with the extension of fermentation time, the mixed strains continued to degrade proteins >10 kDa and those in the 3–10 kDa fraction. This increased the content of proteins <3 kDa to more than 90% (Figure 2D). The <1 kDa fraction increased to a maximum value of 86.82% at 18 h, followed by a small decrease to 85.69% by 20 h. This indicates that fermentation had successfully degraded most large proteins into small peptides or amino acids. According to studies, peptides with small molecules are more chemically stable and have better biological activities (29).

**Table 2:** Molecular weight distribution at different fermentation times.

Fermentation time	Molecular mass (%)			
	<1 kDa	1–3 kDa	3–10 kDa	>10 kDa
17 h	82.99	7.08	7.62	2.3
18 h	86.82	6.7	5.26	1.21
19 h	86.62	7.12	5.35	0.92
20 h	85.69	7.43	5.97	0.91

### 3.7 Amino acids content

The amino acid content exhibited a trend of increase and then decrease with fermentation. The highest total amino acid content was 1.963 mg/mL at 19 h, and a small decrease was observed at 20 h. The decrease was observed possibly because lactic acid bacteria require nutritional factors, such as amino acids, minerals, and vitamins, in a nutrient-rich environment to grow and reproduce (30). Essential amino acids and hydrophobic amino acids reached maximum values of 0.533 and 0.334 mg/mL, respectively, at this time. Among the types of amino acids obtained from the sea buckthorn seed meal, the contents of Glu and Arg were relatively high, with their contents being >0.2 mg/mL. The content of Asp., Ser, and Lys was approximately 0.1 mg/mL. Glu contributes to the synthesis of erythrocytes, proteins, and other amino acids in humans. It can help activate the mind and improve the nutritional environment of brain cells (31). Arg is one of the medicinal amino acid compositions. It has various roles. In addition to being a component of protein synthesis, it can also promote hormone secretion and insulin production and enhance cellular immune function (32). Asp can promote the synthesis of urea from oxygen and carbon dioxide, delay bone damage, eliminate fatigue, protect the liver, and play a role in the lungs to adjust the respiratory effect. Different types of amino acids have different biological effects (33). The presence of these valuable amino acids is one of the crucial reasons for the high value attached to the sea buckthorn seed meal peptide. Thus, 19 h is the best fermentation time for the sea buckthorn seed meal peptide (Table 3).

**Table 3:** Amino acid content at different fermentation times.

Name/(mg/mL)	17 h	18 h	19 h	20 h
Asp	0.130	0.165	0.182	0.145
Glu	0.534	0.669	0.665	0.551
Ser	0.095	0.118	0.123	0.117
Gly	0.080	0.095	0.083	0.078
Arg	0.203	0.242	0.244	0.199
Ala	0.097	0.115	0.088	0.092
Pro	0.033	0.042	0.045	0.036
His	0.040	0.053	0.072	0.045
Thr	0.077	0.096	0.102	0.084
Tyr	0.020	0.030	0.038	0.024
Cys	0.003	0.004	0.006	0.004
Val	0.031	0.042	0.046	0.036
Met	0.007	0.009	0.010	0.008
Phe	0.024	0.032	0.040	0.032
Ile	0.023	0.032	0.037	0.029
Leu	0.034	0.052	0.068	0.050
Lys	0.111	0.128	0.114	0.099
Total amino acids	1.542	1.924	1.963	1.629

### 3.8 Detection of inhibition rates of different molecular weight fractions and amino acid sequencing

**Figure 3:**
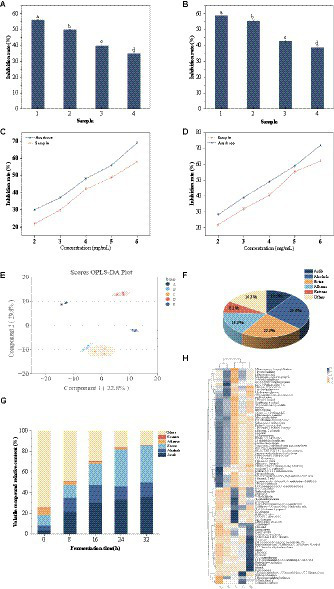
Volatile compound dynamics and *in vitro* hypoglycaemic activity. **(A,B)** Inhibition of α-glucosidase and α-amylase by different fractions at a concentration of 5.0 mg/mL. (1) acarbose, (2) peptide of <1 kDa fraction, (3) peptide of 1–3 kDa fraction, (4) peptide of >3 kDa fraction, **(A)** α-glucosidase, **(B)** α-amylase. **(C)** Determination of α-glucosidase inhibition by different concentrations of >1 kDa fractions. **(D)** Determination of α-amylase inhibition by different concentrations of >1 kDa fractions. **(E)** OPLS-DS score plots. **(A)** 0 h, **(B)** 8 h, **(C)** 16 h, **(D)** 24 h, **(E)** 32 h; **(F)** Plot of the percentage of volatile compounds species; **(G)** Column stacking plot of the relative percentage of volatile compounds; **(H)** Hierarchical cluster analysis of volatile compounds. Rows: different fermentation times, columns: volatile compounds [these values are expressed as mean of three replicates ± standard deviation (SD), lower case letters represent significance, *p* < 0.05].

*In vitro* hypoglycemic activity of the sea buckthorn seed peptides of <1 kDa, 1–3 kDa, and >3 kDa fractions was detected through ultrafiltration. At 5 mg/mL, the <1 kDa fraction of the sea buckthorn seed meal peptide exhibited the best inhibitory activity against α-glucosidase and α-amylase, which were 49.87% ± 0.37 and 55.56% ± 0.51%, respectively (Figures 3A,B), followed by the peptides of 1–3 kDa fractions. Subsequently, inhibition rate tests were conducted on different concentrations of <1 kDa components. The IC_50_ values for α-glucosidase and α-amylase of the <1 kDa components were found to be 5.04 mg/mL and 4.65 mg/mL, respectively (Figures 3C,D). α-Glucosidase catalyzes carbohydrate digestion. Hydrolysis of both starch and disaccharides produces glucose, which causes an increase in blood glucose levels (34). α-Amylase catalyzes the hydrolysis of starch, glycogen, and α-1,4-glycosidic bonds to monosaccharides (35). The <1 kDa fraction inhibited these two enzymes possibly because hydrophobic amino acids in the sea buckthorn seed meal peptides are the most abundant by 19 h of fermentation. The catalytic effect of the enzyme is affected by the binding site of the enzyme or that of the substrate (36). Obaroakpo et al., found that the higher the content of hydrophobic amino acids, the higher the α-glucosidase inhibitory activity of the protein in the fermented quinoa protein drink. This provides a basis for the development of novel hypoglycemic drugs (37). In addition, the contents of glutamic acid, arginine, lysine, serine, and glycine in the sea buckthorn seed meal peptide were higher. This may be among the reasons for the inhibitory effect noted on these two enzymes.

The determination results of <1 kDa sea buckthorn seed meal peptides were compared with the sea buckthorn NCBI database, identifying 18 peptide sequences. The total number of amino acids in these peptides ranged from 5 to 9 (Table 4), and all peptides had a molecular weight below 1 kDa, with hydrophobicity ranging from 29 to 80%. Moreover, peptides with a molecular weight below 600 Da exhibited relatively higher hydrophobicity. These results indicate that the selected peptide segments all contain hydrophobic amino acids, demonstrating a certain correlation between hydrophobic amino acids and hypoglycemic activity. Based on the predicted hydrophobicity and biological activity, the peptides FLVAG and FYLPKM were preliminarily selected. These two peptides exhibited superior hydrophobicity and biological activity compared to the other peptides. Studies have shown that amino acid sequences containing leucine, proline, tyrosine, and valine can participate in blood sugar control, enhancing the hypoglycemic activity of peptides (38). Considering these factors, we chose the peptide FYLPKM for further research, as it may be an advantageous peptide for hypoglycemic activity.

**Table 4:** Amino acid sequence results for the <1 kDa fraction.

No.	Amino acid sequences	Molecular weight (Da)	*m/z*	Isoelectric point	Hydrophobicity (%)	Bioactivity
1	GAISL	459.269	460.277	5.88	60	0.3249
2	QDLFFS	755.350	756.355	3.8	50	0.7298
3	PICNSLGPL	913.458	914.471	5.85	44	0.7791
4	KNSDLFLL	949.512	475.761	6.19	50	0.7485
5	MTGLPDI	745.368	746.372	3.8	43	0.2518
6	ECITLGIPT	927.474	928.489	4.0	44	0.2598
7	AEFLT	579.290	580.298	4.0	60	0.3038
8	FLVAG	505.290	506.294	5.88	80	0.5430
9	DPFATP	646.296	647.304	3.8	33	0.7255
10	ILGYTEE	823.396	824.405	3.8	29	0.066
11	VLSSAM	622.300	623.303	5.88	67	0.1886
12	WLYNGG	708.323	709.331	5.88	33	0.7640
13	VFSGL	521.285	522.292	5.88	60	0.5920
14	LAVHI	593.354	594.363	7.1	80	0.1231
15	IVGNVF	647.364	648.372	5.88	67	0.2757
16	EADQFR	765.329	766.336	4.38	33	0.3395
17	FYLPKM	797.415	399.719	8.94	50	0.9012
18	EGKAPI	613.344	614.351	6.35	33	0.1646

### 3.9 Detection of volatile compounds with different fermentation time

Lactic acid fermentation can increase the flavor compounds, produce organic acids, and enhance the nutritional and sensory properties (e.g., antioxidant capacity, color) of the substrate (39). OPLS-DA was used to analyze the volatile components of sea buckthorn seed meal peptide solution at fermentation times of 0 h, 8 h, 16 h, 24 h, and 32 h. As shown in Figure 3E, the model had good explanatory and predictive capabilities with *Q*^2^ = 0.908, *p* < 0.005 and *R*^2^*Y* = 0.981, *p* < 0.005. Based on VIP > 1, 86 volatile compounds were screened out (Supplementary Table S1), including 9 acids, 22 alcohols, 20 esters, 14 alkanes, 7 ketones, and 14 other compounds. The proportions of each compound are shown in Figure 3F.

The relative content of volatile compounds in sea buckthorn seed meal varied at different fermentation stages. With the extension of fermentation time, the relative content of volatile flavor compounds increased, consistent with the results of Suo et al. (40). Mixed-strain fermentation activated the hexose kinase pathway of the phosphogluconate pathway, leading to an increase in acid content. Acetyl-CoA and alcohols produced esters under the action of esterase or acyltransferase (41). The relative content of acids and esters increased significantly from 3 and 10% to 33 and 36% (0–24 h), respectively. It is worth noting that the relative content of acetic acid significantly increased from 0 to 0.057 μg/mL (0–24 h), contributing greatly to the aroma of sea buckthorn seed meal peptide solution (Figure 3G). In addition, Hexanoic acid, 2-phenylethyl ester and Formic acid, heptyl ester imparted fruity and rose aromas to the solution (42). Importantly, the relative content of acids and esters remained at 36% until the end of fermentation, which may play a positive role in the aroma characteristics of mixed-strain fermented sea buckthorn seed meal peptide solution.

The relative content of alcohol substances increased from 5 to 17% (0–16 h). This is because the fermentation of the strain causes the protein in the raw material to break down into amino acids, which then undergo deamination and decarboxylation reactions to generate alcohol substances (43). The production of alcohol substances such as 1-Heptanol, 1-Hexanol, 1-Hexanol, 2-ethyl- increased the floral and fruity aroma. Subsequently, some alcohols and carboxylic acids react to form esters, mostly in the form of methyl esters (44). In our study, we also found a significant increase in the relative content of Damascenone and Ionone, which impart fruity, sweet, woody, and floral notes to the seabuckthorn seed meal peptide liquid (45). They may have played a positive role in enhancing the overall sensory properties of seabuckthorn seed meal. The results of hierarchical cluster analysis (HCA) are consistent with the changes in the above-mentioned volatile compounds (Figure 3H).

## 4 Conclusion

This study prepared seabuckthorn seed meal peptide through mixed fermentation, increasing the utilization value of seabuckthorn seed meal. The research indicates that maltose and MgSO_4_·7H_2_O supplemented the necessary nutrients for fermentation. When the fermentation time was 19 h, a mixed fermentation of *L. fermentum*, *B. subtilis*, *L. case*, *L. reuteri*, and *L. acidophilus* degraded 43.85% of the protein in seabuckthorn seed meal into small molecule peptides. At this time, the peptide molecular weight was mainly distributed below 3 kDa, and the amino acid content reached its maximum value, demonstrating that mixed strain fermentation is beneficial for the degradation of seabuckthorn protein. At a peptide concentration of 5 mg/mL, peptides with a molecular weight of <1 kDa showed the best inhibition activity against α-amylase and α-glucosidase, possibly due to the amino acid sequence of FYLPKM. Additionally, during the fermentation process, the content of acids and esters significantly increased from 3 and 10% to 33 and 36% (0–24 h). This change had a certain positive effect on the aroma characteristics of the mixed fermentation seabuckthorn seed meal peptide liquid and provided a theoretical basis for the application of seabuckthorn seed meal peptide in food. Subsequent work may focus on the synthesis, functional verification, hypoglycemic mechanism, and peptide beverage research of sea buckthorn seed meal peptides.

## Data availability statement

The original contributions presented in the study are included in the article/Supplementary material, further inquiries can be directed to the corresponding author.

## Author contributions

JY: Formal analysis, Methodology, Project administration, Writing – original draft, Writing – review & editing. JH: Writing – review & editing, Writing – original draft. AA: Writing – review & editing. YM: Data curation, Methodology, Writing – original draft. XY: Writing – review & editing. XL: Data curation, Writing – original draft. MZ: Writing – review & editing. LW: Funding acquisition, Writing – review & editing.

## Footnotes

^1^ http://distilldeep.ucd.ie/PeptideRanker/

^2^ https://webs.iiitd.edu.in/raghava/toxinpred/design.php
