# Supplementary material for: Preparation of sea buckthorn (Hippophae rhamnoides L.) seed meal peptide by mixed fermentation and its effect on volatile compounds and hypoglycemia
Source: Front Nutr. 2024 Feb 13;11:1355116. doi: 10.3389/fnut.2024.1355116 (PMC10896959; doi:10.3389/fnut.2024.1355116)
Supplement: Supplementary file 1 [file Data_Sheet_1.docx]

**Supplementary material:**

**Supplementary Table 1** Volatile compounds content of sea buckthorn seed meal fermentation broth at different times.

| No. | Compound | CAS | RT  (min) | Concentration（μg/mL） | | | | |
| --- | --- | --- | --- | --- | --- | --- | --- | --- |
|  |  |  |  | 0 hours | 8 hours | 16 hours | 24 hours | 32 hours |
| Acid | | | | | | | | |
| 1 | Cyclohexan-1,4,5-triol-3-one-1-carboxylic acid | 105735-75-9 | 3.74 | n.c | 0.0061±0.0003b | 0.0167±0.0019a | 0.0015±0.0002bc | 0.0187±0.0053a |
| 2 | N-Methyltaurine | 107-68-6 | 2.99 | n.d | 0.0287±0.0013c | 0.0543±0.005a | 0.0376±0.0031b | 0.0298±0.0037c |
| 3 | Acetic acid, oxo- | 924-44-7 | 3.74 | n.c | 0.0001±0.0001bc | 0.0003±0.0001b | 0.0002±0.0001bc | 0.009±0.0001a |
| 4 | D-Alanine | 338-69-2 | 3.38 | 0.0002±0c | 0.0005±0.0001c | 0.0014±0.0002c | 0.0256±0.0015a | 0.0059±0.0011b |
| 5 | dl-Threonine | 80-68-2 | 9.39 | n.c | 0.0001±0c | 0.0002±0c | 0.0011±0.0001b | 0.0022±0.0003a |
| 6 | Pterin-6-carboxylic acid | 948-60-7 | 11.58 | 0.0031±0.0001a | 0.0027±0.0002b | 0.0012±0c | 0.0006±0.0002d | n.e |
| 7 | Acetic acid | 64-19-7 | 4.92 | n.d | 0.0025±0.0001d | 0.0124±0.0013c | 0.0574±0.0029b | 0.0673±0.0021a |
| 8 | N-Methylglycine | 6414-57-9 | 2.21 | 0.0001±0d | 0.0004±0.0001cd | 0.0008±0.0001c | 0.0038±0.0006b | 0.0046±0.0001a |
| 9 | N-(3,5-Dinitropyridin-2-yl)-L-aspartic acid | 35899-60-6 | 5.5 | n.d | 0.0001±0cd | 0.0002±0c | 0.0007±0.0002b | 0.0012±0.0001a |
| Alcohol | | | | | | | | |
| 10 | Z,Z-2,5-Pentadecadien-1-ol | 139185-79-8 | 26.44 | n.c | 0.0008±0.0001a | 0.0002±0.0001b | 0.0001±0bc | n.c |
| 11 | 1-Heptanol | 111-70-6 | 11.93 | n.c | n.bc | 0.0001±0b | 0.0003±0.0001a | 0.0001±0.0001bc |
| 12 | DL-Alaninol | 6168-72-5 | 3.63 | n.c | 0.0007±0.0002bc | 0.0015±0.0001b | n.c | 0.0147±0.0013a |
| 13 | Panaxydol | 72800-72-7 | 30.41 | n.c | 0.0002±0.0001bc | 0.0002±0b | 0.0021±0.0002a | 0.0002±0.0001bc |
| 14 | 1-Heptatriacotanol | 105794-58-9 | 26.42 | 0.0003±0.0001b | 0.0006±0.0001a | 0.0005±0.0001a | 0.0002±0.0001b | n.c |
| 15 | (R)-(-)-2-Amino-1-propanol | 35320-23-1 | 2.17 | 0.0001±0d | 0.0153±0.0003c | 0.0292±0.0022b | 0.0012±0.0001d | 0.0328±0.0001a |
| 16 | Cyclopropyl carbinol | 1219805-67-0 | 4.78 | 0.0003±0.0001c | 0.0011±0.0001b | 0.0019±0.0002a | 0.0011±0.0002b | 0.0011±0.0001b |
| 17 | Cyclobutanol | 2919-23-5 | 4.35 | 0.0001±0c | 0.0027±0.0003b | 0.0096±0.0006a | 0.0097±0.0017a | 0.0018±0.0001b |
| 18 | 10-Azido-1-decanethiol | 57395-48-9 | 8.01 | n.b | n.b | n.b | n.b | 0.0004±0.0003a |
| 19 | 1-Octanol, 2-butyl- | 3913-02-8 | 26.43 | 0.0002±0a | 0.0002±0.0001ab | 0.0001±0.0001abc | n.bc | n.c |
| 20 | 2,6-Octadien-1-ol,2,7-dimethyl | 32663-38-0 | 14.22 | n.c | 0.0001±0.0001bc | 0.0001±0.0001bc | 0.0005±0.0001a | 0.0002±0b |
| 21 | 1-Heptanol, 2-propyl- | 10042-59-8 | 17.35 | 0.0002±0a | 0.0002±0.0001ab | 0.0001±0.0001abc | n.bc | n.c |
| 22 | 1-Hexanol | 111-27-3 | 7.99 | n.d | 0.0017±0.0004c | 0.0031±0.0002b | 0.004±0.0006a | 0.0018±0.0003c |
| 23 | 1,2,6-Hexanetriol | 106-69-4 | 6.4 | 0.0002±0a | 0.0002±0.0001ab | 0.0001±0.0001abc | n.bc | n.c |
| 24 | 9-Oxabicyclo[6.1.0]nonan-4-ol | 2616-81-1 | 17.88 | 0.0002±0a | 0.0002±0.0001ab | 0.0001±0.0001abc | n.bc | n.c |
| 25 | 1-Hexadecanol,2-methyl | 2490-48-4 | 15.64 | 0.0004±0a | 0.0003±0.0001a | 0.0001±0c | 0.0002±0.0001b | n.d |
| 26 | Ethanol | 64-17-5 | 2.95 | n.b | 0.0007±0.0001b | 0.0009±0.0002b | 0.0146±0.0019a | 0.0007±0.0001b |
| 27 | 2-Hexyl-1-octanol | 19780-79-1 | 25.83 | 0.0006±0.0001a | 0.0005±0.0001ab | 0.0004±0.0001bc | 0.0003±0.0001c | 0.0001±0.0001d |
| 28 | Diglycolamine | 929-06-6 | 3.21 | n.b | 0.0007±0.0001b | 0.0009±0.0002b | 0.0146±0.0019a | 0.0007±0.0001b |
| 29 | 1-Hexanol, 2-ethyl- | 104-76-7 | 14.47 | 0.0013±0.0001a | 0.0009±0.0001b | 0.0004±0c | 0.0001±0d | n.e |
| 30 | 2-Propyl-1-pentanol | 58175-57-8 | 14.45 | 0.0012±0.0001a | 0.0006±0.0001b | 0.0002±0.0001c | 0.0001±0.0001d | n.d |
| 31 | Falcarinol | 21852-80-2 | 27.03 | n.c | n.bc | 0.0001±0b | 0.0003±0.0001a | 0.0001±0.0001bc |
| Ester | | | | | | | | |
| 32 | 10-Heptadecen-8-ynoicacid, methyl ester, (E)- | 16714-85-5 | 17.93 | n.b | n.b | n.b | 0.0001±0a | n.b |
| 33 | heptyl formate | 112-23-2 | 11.99 | 0.0001±0c | n.c | 0.0006±0.0001b | n.c | 0.0031±0.0004a |
| 34 | Ethyl iso-allocholate | 15073-99-1 | 21.89 | 0.0004±0.0001a | 0.0005±0.0001a | 0.0002±0b | 0.0001±0.0001b | n.b |
| 35 | 2-Phenylethyl hexanoate | 6290-37-5 |  | 0.0012±0c | 0.0016±0.0002c | 0.0024±0.0003b | 0.0012±0.0002c | 0.0046±0.0002a |
| 36 | Carbonic acid, ethylphenylmethyl ester | 22768-02-1 | 35.14 | 0.0002±0a | 0.0002±0.0001ab | 0.0001±0.0001abc | n.bc | n.c |
| 37 | Ethyl(6Z,9Z,12Z)-6,9,12-octadecatrienoate | 31450-14-3 | 26.43 | 0.0002±0a | 0.0002±0.0001ab | 0.0001±0.0001abc | n.bc | n.c |
| 38 | ethyl(2S)-2-aminopropanoate | 3082-75-5 | 3.09 | 0.0002±0a | 0.0002±0.0001ab | 0.0001±0.0001abc | n.bc | n.c |
| 39 | tetradecan-2-yl propanoate | 959010-56-1 | 15.84 | 0.0003±0.0001a | n.b | n.b | n.b | n.b |
| 40 | (z)-7-tetradecen-1-yl acetate | 16974-10-0 | 12.75 | 0.0005±0.0001a | 0.0004±0.0001a | 0.0003±0.0001a | 0.0001±0.0001b | n.b |
| 41 | Valeric acid, 3-tridecyl ester | 55044-82-1 | 6.57 | 0.0007±0a | 0.0006±0.0001a | 0.0001±0bc | 0.0002±0.0001b | n.c |
| 42 | 3-Chloropropionic acid,2-chlorophenyl ester | 213008-02-7 | 21.53 | 0.0002±0a | 0.0001±0.0001ab | 0.0001±0.0001ab | n.b | n.b |
| 43 | (R)-lavandulyl acetate | 20777-39-3 | 17.7 | n.b | n.b | n.b | 0.0001±0.0001b | 0.0002±0.0001a |
| 44 | Formic acid,2-ethylhexyl ester acid, methylester | 5460-45-7 | 14.47 | 0.0002±0a | 0.0002±0.0001ab | 0.0001±0.0001abc | n.bc | n.c |
| 45 | Dithiocarbamate,S-methyl-,N-(2-methyl-3-oxobutyl)- | 135923-14-7 | 33.7 | 0.0004±0.0001a | 0.0003±0.0002ab | 0.0001±0.0001bc | n.bc | n.c |
| 46 | sec-Butyl nitrite | 924-43-6 | 4.33 | 0.0027±0.0001b | 0.003±0.0001a | 0.002±0.0002 | 0.0008±0.0001d | 0.0002±0.0001e |
| 47 | 10-Undecenoic acid,octyl ester | 28080-85-5 | 17.7 | 0.0004±0.0001a | 0.0004±0.0001a | 0.0001±0.0001b | n.bc | n.c |
| 48 | Ethyl caprylate | 106-32-1 | 22.26 | 0.0005±0.0001a | 0.0004±0.0001ab | 0.0003±0.0001b | 0.0001±0.0001c | n.c |
| 49 | Formyl acetate | 2258-42-6 | 4.54 | 0.0006±0.0001d | 0.0055±0.0002d | 0.0349±0.0033c | 0.0843±0.0047a | 0.0685±0.0012b |
| 50 | Methyl glyoxylate | 922-68-9 | 3.8 | 0.0003±0.0001e | 0.0096±0.0004d | 0.0183±0.0018c | 0.0495±0.0028b | 0.0607±0.0016a |
| 51 | Nonanoic acid,2,4,6-trimethyl-, methylester, (R,R,R)-(-)- | 2490-57-5 | 22.27 | 0.0007±0a | 0.0005±0.0001b | 0.0002±0.0001c | n.d | n.d |
| Alkane | | | | | | | | |
| 52 | (+)-2-Aminoheptane | 33758-16-6 | 4.35 | n.c | 0.0006±0.0001c | 0.0015±0.0002b | 0.0036±0.0006a | 0.0002±0.0001c |
| 53 | dodecan-2-yl 2,2,2-trifluoroacetate | 1894-68-4 | 22.27 | 0.0012±0.0001a | 0.0002±0.0001bc | 0.0002±0.0001bc | n.c | 0.0004±0.0002b |
| 54 | Heneicosane,11-(1-ethylpropyl)- | 55282-11-6 | 17.35 | 0.0003±0.0001b | 0.0006±0.0001a | 0.0005±0.0001a | 0.0002±0.0001b | n.c |
| 55 | 2-bromododecane | 13187-99-0 | 15.61 | 0.0006±0.0001a | 0.0002±0.0004ab | 0.0002±0.0003ab | 0.0001±0ab | n.b |
| 56 | 10-Methylnonadecane | 56862-62-5 | 35.15 | 0.0002±0.0001a | 0.0002±0.0001a | 0.0002±0.0001a | 0.0001±0ab | n.b |
| 57 | 9-n-Hexylheptadecane | 55124-79-3 | 27.85 | 0.0002±0a | 0.0002±0.0001ab | 0.0001±0.0001abc | n.bc | n.c |
| 58 | 1,1-didodecoxyhexadecane | 56554-64-4 | 17.94 | 0.0002±0a | 0.0002±0.0001ab | 0.0001±0.0001abc | n.bc | n.c |
| 59 | Hexane, 2,3,5-trimethyl- | 1069-53-0 | 6.4 | 0.0002±0a | 0.0002±0.0001ab | 0.0001±0.0001abc | n.bc | n.c |
| 60 | 1,2-Epoxynonane | 130466-96-5 | 15.88 | 0.0002±0a | 0.0002±0.0001ab | 0.0001±0.0001abc | n.bc | n.c |
| 61 | Dodecane | 112-40-3 | 35.14 | 0.0002±0a | 0.0002±0.0001ab | 0.0001±0.0001abc | n.bc | n.c |
| 62 | nitrosomethane | 865-40-7 | 2.79 | n.a | n.a | n.a | n.a | n.a |
| 63 | 1-cyclopropylpentane | 2511-91-3 | 11.99 | 0.0014±0b | 0.0026±0.0001a | 0.0015±0.0002b | 0.0002±0.0001c | n.c |
| 64 | Octadecane, 6-methyl- | 10544-96-4 | 15.63 | 0.0005±0.0001a | 0.0004±0.0001a | 0.0003±0.0001a | 0.0001±0.0001b | n.b |
| 65 | Decane,2,3,5,8-tetramethyl | 192823-15-7 | 35.14 | 0.0013±0a | 0.0011±0.0001b | 0.0008±0c | 0.0002±0.0001d | n.e |
| Ketone | | | | | | | | |
| 66 | Ionone | 8013-90-9 | 25.76 | n.c | n.bc | 0.0001±0b | 0.0003±0.0001a | 0.0001±0.0001bc |
| 67 | Damascenone | 23696-85-7 | 30.4 | 0.0001±0.0001b | 0.0002±0.0001b | 0.0003±0.0001ab | 0.0006±0.0001a | 0.0004±0.0004ab |
| 68 | 8-Hydroxy-2-octanone | 25368-54-1 | 12.74 | 0.0002±0a | 0.0002±0.0001ab | 0.0001±0.0001abc | n.bc | n.c |
| 69 | 3,4,5-Trimethyldihydrofuran-2-one | 70358-85-9 | 8.01 | n.c | n.bc | 0.0001±0b | 0.0003±0.0001a | 0.0001±0.0001bc |
| 70 | 2-Hexanone,4-hydroxy-3-propyl | 62338-17-4 | 12.74 | 0.0005±0.0001a | 0.0004±0.0001a | 0.0003±0.0001a | 0.0001±0.0001b | n.b |
| 71 | 3-Hydroxymethylene-1,7,7-trimethylbicyclo[2.2.1]heptan-2-one | 20618-41-1 | 28.57 | 0.0002±0a | 0.0002±0.0001ab | 0.0001±0.0001abc | n.bc | n.c |
| 72 | 9-Oxabicyclo[3.3.1]nonan-2-one, 6-hydroxy | 35570-54-8 | 15.61 | n.d | 0.0001±0.0001cd | 0.0003±0.0001bc | 0.0006±0.0001a | 0.0005±0.0002ab |
| Other | | | | | | | | |
| 73 | Dextroamphetamine | 51-64-9 | 4.61 | n.c | 0.0006±0.0001bc | 0.0105±0.0011a | 0.0005±0.0001bc | 0.0011±0.0001b |
| 74 | Hexyl octyl ether | 17071-54-4 | 15.62 | 0.0003±0.0001a | 0.0002±0.0002ab | 0.0001±0.0001ab | n.b | 0.0001±0.0001ab |
| 75 | 1-Octene, 3,7-dimethyl- | 4984-01-4 | 11.99 | 0.0001±0c | 0.001±0.0001c | 0.0114±0.0013a | 0.0004±0.0001c | 0.0041±0.0008b |
| 76 | 4-Fluorohistamine | 49872-60-8 | 3.77 | 0.0001±0bc | n.c | n.c | 0.0003±0.0002ab | 0.0004±0.0001a |
| 77 | 1,3-Cyclopentadiene,1,2,3,4-tetramethyl-5-methylene | 76089-59-3 | 18.49 | 0.0001±0ab | 0.0001±0a | 0.0001±0ab | n.c | n.bc |
| 78 | 1-Octadecanesulphonyl chloride | 10147-41-8 | 25.84 | 0.0003±0.0001b | 0.0006±0.0001a | 0.0005±0.0001a | 0.0002±0.0001b | n.c |
| 79 | Methylpent-4-enylamine | 5831-72-1 | 4.33 | 0.0003±0d | 0.0011±0.0001c | 0.0017±0.0001b | 0.0052±0.0004a | 0.0009±0.0002c |
| 80 | Z,Z,Z-1,4,6,9-Nonadecatetraene | 169900-26-9 | 17.7 | n.d | 0.0004±0a | 0.0003±0b | 0.0002±0c | 0.0002±0c |
| 81 | 1-hydroperoxyhexane | 4312-76-9 | 7.93 | 0.0013±0d | 0.0025±0.0002b | 0.0021±0.0001c | 0.0047±0.0001a | 0.0024±0.0001bc |
| 82 | 2-Pyridinamine,5-methyl-N-nitro- | 7464-15-5 | 7.1 | 0.0009±0b | 0.0012±0.0001a | 0.0009±0.0001b | 0.0009±0.0002b | 0.0005±0.0001c |
| 83 | Benzeneethanamine,2,5-difluoro-á,3,4-trihydroxy-N-methyl | 152434-78-1 | 16.61 | 0.0016±0.0001ab | 0.0019±0.0001a | 0.0014±0.0002bc | 0.0011±0.0001d | 0.0013±0.0002cd |
| 84 | (2S)-2-amino-N-ethylpropanamide | 71773-95-0 | 2.17 | 0.004±0.0001a | 0.0041±0.0006a | 0.0039±0.0003a | 0.0038±0.0001a | 0.0027±0.0002b |
| 85 | Azulene | 275-51-4 | 21.48 | n.d | 0.0031±0.0002c | 0.0055±0.0011b | 0.0114±0.0004a | 0.0032±0.0002c |
| 86 | (2-Aziridinylethyl)amine | 4025-37-0 | 2.01 | 0.0658±0.0002b | 0.0783±0.0031a | 0.0463±0.0046c | 0.0337±0.002d | 0.0356±0.0042d |

**Supplementary Table 2** Basic chemical composition of sea buckthorn seed meal.

|  | Protein | Moisture | Oil | Ash | Carbohydrates |
| --- | --- | --- | --- | --- | --- |
| Content (%) | 45% | 5.5% | 8.2% | 4.8% | 11.8% |
